# Supplementary material for: The interactome of intact mitochondria by cross-linking mass spectrometry provides evidence for coexisting respiratory supercomplexes
Source: Mol Cell Proteomics. 2017 Dec 8;17(2):216–32. doi: 10.1074/mcp.RA117.000470 (PMC5795388; doi:10.1074/mcp.RA117.000470)
Supplement: Supplemental Data [file supp_RA117.000470_133922_0_supp_23978_fzffwf.zip › spectra_annotation/mito_spectra_annotation/390-1-5-1-9-1.pdf]

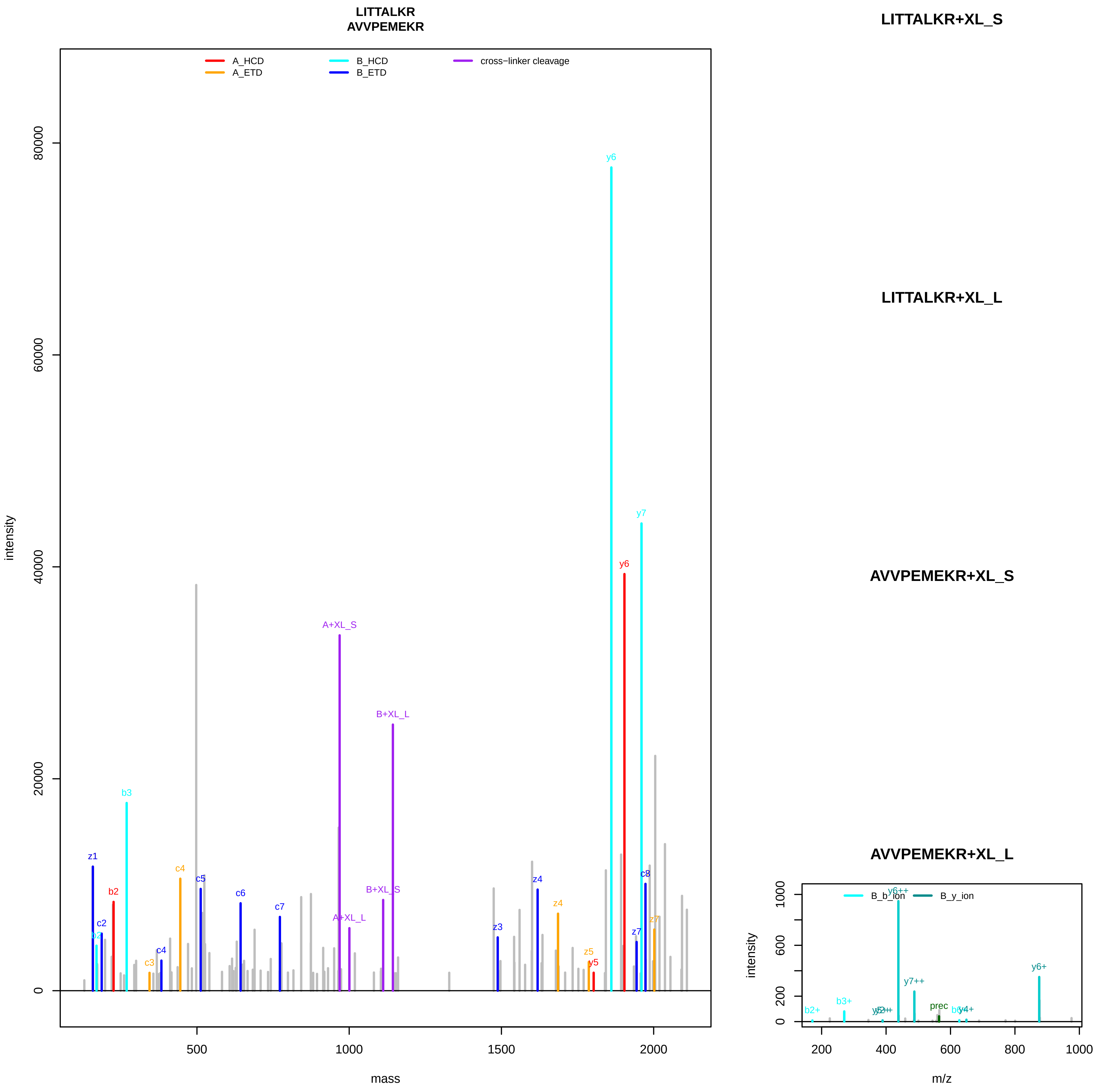
LITTALKR
AVVPEMEKR
A_HCD
A_ETD
B_HCD
B_ETD
cross−linker cleavage
intensity
mass
0
20000
40000
60000
80000
500
1000
1500
2000
y6
y7
y6
A+XL_S
B+XL_L
B+XL_S
A+XL_L
b3
z1
c2
b2
c3
c4
c5
c6
c7
z3
z4
z4
z5
y5
z7
c8
z7
LITTALKR+XL_S
LITTALKR+XL_L
AVVPEMEKR+XL_S
AVVPEMEKR+XL_L
B_b_ion
B_y_ion
y6++
y7++
y6+
b2+
b3+
y4+
prec
m/z
0
200
600
1000
200
400
600
800
1000
